# New Networks, New Connections: Geriatric Student Scholars Embrace Adaptive Learning

**DOI:** 10.1093/geroni/igab046.1834

**Published:** 2021-12-17

**Authors:** Robin McAtee

**Affiliations:** University of Arkansas for Medical Sciences, Little Rock, Arkansas, United States

## Abstract

The Arkansas Geriatric Education Collaborative (AR’s GWEP) embraces, nurtures, and encourages students with a passion for caring for older adults. Each year five geriatric scholars are chosen from across the spectrum of health services schools (MD, RN, PT, PA, Pharm D, dental hygiene, etc.) to enhance their geriatric knowledge and experience. Requirements focus on geriatric academic and community-based opportunities. However, these opportunities drastically changed with the pandemic. Therefore, the students became very innovative as they trudged forward to meet and exceed the scholar objectives. They participated in various virtual events to fulfil their academic and community participation requirements. They worked together to develop and implement an interdisciplinary final project that marketed to and engaged rural isolated older adults in a fun educational event aimed at preventing social isolation in older adults and caregivers. Students learned how to connect to and bridge the digital divide with isolated rural older adults.

